# Haematology and Serum Biochemistry Parameters and Variations in the Eurasian Beaver (*Castor fiber*)

**DOI:** 10.1371/journal.pone.0128775

**Published:** 2015-06-12

**Authors:** Simon J. Girling, Roisin Campbell-Palmer, Romain Pizzi, Mary A. Fraser, Jonathan Cracknell, Jon Arnemo, Frank Rosell

**Affiliations:** 1 Veterinary Department, Living Collection, Royal Zoological Society of Scotland, Edinburgh, United Kingdom; 2 Faculty of Arts and Sciences, Department of Environmental and Health Studies, Telemark University College, Bø i Telemark, Norway; 3 Conservation Programmes, Royal Zoological Society of Scotland, Edinburgh, United Kingdom; 4 School of Life, Sport and Social Science, Edinburgh Napier University, Sighthill Campus, Edinburgh, United Kingdom; 5 Longleat Safari and Adventure Park, Longleat, Warminster, Wiltshire, United Kingdom; 6 Department of Forestry and Wildlife Management, Faculty of Applied Ecology and Agricultural Sciences, Hedmark University College, Campus Evenstad, Koppang, Norway; 7 Department of Wildlife, Fish and Environmental Studies, Faculty of Forest Sciences, Swedish University of Agricultural Sciences, Umeå, Sweden; Institut Pluridisciplinaire Hubert Curien, FRANCE

## Abstract

Haematology parameters (N = 24) and serum biochemistry parameters (N = 35) were determined for wild Eurasian beavers (*Castor fiber*), between 6 months – 12 years old. Of the population tested in this study, N = 18 Eurasian beavers were from Norway and N = 17 originating from Bavaria but now living extensively in a reserve in England. All blood samples were collected from beavers via the ventral tail vein. All beavers were chemically restrained using inhalant isoflurane in 100% oxygen prior to blood sampling. Results were determined for haematological and serum biochemical parameters for the species and were compared between the two different populations with differences in means estimated and significant differences being noted. Standard blood parameters for the Eurasian beaver were determined and their ranges characterised using percentiles. Whilst the majority of blood parameters between the two populations showed no significant variation, haemoglobin, packed cell volume, mean cell haemoglobin and white blood cell counts showed significantly greater values (p<0.01) in the Bavarian origin population than the Norwegian; neutrophil counts, alpha 2 globulins, cholesterol, sodium: potassium ratios and phosphorus levels showed significantly (p<0.05) greater values in Bavarian versus Norwegian; and potassium, bile acids, gamma globulins, urea, creatinine and total calcium values levels showed significantly (p<0.05) greater values in Norwegian versus Bavarian relict populations. No significant differences were noted between male and female beavers or between sexually immature (<3 years old) and sexually mature (≥3 years old) beavers in the animals sampled. With Eurasian beaver reintroduction encouraged by legislation throughout Europe, knowledge of baseline blood values for the species and any variations therein is essential when assessing their health and welfare and the success or failure of any reintroduction program. This is the first study to produce base-line blood values and their variations for the Eurasian beaver.

## Introduction

Eurasian beavers (*Castor fiber*) are large (adults >20 kg), herbivorous, semi-aquatic, territorial, monogamous rodents that live in small family groups and reach sexual maturity around 3 years of age [1]. The Eurasian beaver was previously widespread throughout fresh water habitats of Europe and Asia [2]. However, by the 20^th^ century the species had been reduced to approximately 1,200 individuals in eight isolated populations, largely through hunting [3–5]. Conservation efforts, including hunting bans and reintroduction programmes have seen beaver numbers recover throughout most of its former range [3]. Over 200 translocations have been recorded, across 25 European countries and current population figures estimate > 1.04 million animals [6]. Previous genetic studies suggest that the current Eurasian beaver population can be divided into two evolutionary significant units (ESU)—the Eastern European and Western European populations [7]. Bavarian beavers are thought to derive from a mixture of the Eastern and Western European ESU; Norwegian beavers are a relict population from the Western European ESU [8, 9]. More recent genetic analysis suggests that such variations represent the two extremes of one species [10].

Current EU legislation promotes reintroduction of beavers and other pre-existing species across their former European ranges (Council Directive 92/43/EEC, ‘Habitats Directive’). With this legislation in place several European countries have started or completed the process of reintroducing Eurasian beavers. In the UK (Scottish Beaver Trial, Knapdale, Scotland, UK) a pilot project has been completed to assess the viability of reintroducing the Eurasian beaver. As part of any responsible reintroduction program or trials, pre-release health assessments, and post release health assessments are essential to ensure health and welfare legislation is complied with. One important method of assessment of health in any animal is to assess haematological and biochemical parameters [11]. It also provides one means by which the suitability of a release location and the level to which the released animals are coping in their habitat can be assessed. Blood haematology and serum chemistry have been used in health assessments for a range of wild and domestic animals and so can be an essential part of investigating the success or failure of reintroduction programs [12, 13]. To develop accurate reference values for haematological and biochemical values, baseline data must first be collected from healthy, free ranging populations [14]. Once this has been established, the health of other populations or individuals can be evaluated by comparing their blood parameters against the standard healthy, wild population.

To date, haematological and serum biochemistry parameters for Eurasian beavers have not been published. Haematological and serum biochemistry values have, however, been published for captive North American beavers (*C*. *canadensis*) [15–19].

This study is the first to provide baseline haematological and serum biochemistry parameters for the Eurasian beaver and to compare across two beaver sub-populations. This information may assist health monitoring and assessment of captive, reintroduced and translocated individuals and is of particular relevance at this time with the proposed reintroduction of the Eurasian beaver to Britain. It will also enable health assessment comparisons between other wild and captive populations of beaver.

## Material and Methods

This study was approved by the Norwegian Animal Research Authority, Norwegian Environment Agency and the Royal Zoological Society of Scotland internal ethics committee. All animals were sampled as part of routine health assessments or in the process of surgical neutering, under general anaesthesia and by qualified veterinary surgeons ensuring preservation of welfare and prevention of any suffering as detailed below.

### Study area and animals

#### Relict Norwegian Beavers

The relict Norwegian beavers used in this study were part of a wild population living in the Telemark region, Norway. Beavers were caught from two main rivers, the Gvarv and the Lunde in August 2010. In total 18 beavers were caught as part of a health survey undertaken by Telemark University College and the Royal Zoological Society of Scotland. This was comprised of 10 males and 8 females with an age range of 6 months to 12 years. Of these beavers, 5 were considered sexually immature (<3 years old) and 13 sexually mature (≥3 years old).

#### Bavarian Beavers

The Bavarian beavers in this study were part of a family group living in an extensive but enclosed area covering 20 hectares in southern England in September 2011. In total 17 beavers were caught as part of a health assessment and reproductive control procedure. This was comprised of 9 males and 8 females with an age range of 6 months to 12 years. Of these beavers, 9 were considered sexually immature (<3 years old) and 8 were considered sexually mature (≥3 years old).

### Sampling

All beavers were live-trapped through netting from a boat as described in Rosell and Hovde [20]. After capture each beaver was fully examined for any signs of poor health or disease. Blood samples for this study were only collected from clinically healthy individuals. Each beaver was morphometrically measured and sex was determined on basis of colour and viscosity of anal gland secretion [21]. Age class (sexually immature i.e. <3 years or sexually mature i.e. ≥3 years old) was determined through records (either from captive collection records of from data collected as part of long term-study) if known or age class estimated from body size and weight.

### Chemical Restraint

The time from capture to induction of anaesthesia for both groups was between 30–90 minutes. All beavers were anaesthetised using 4% isoflurane in 100% oxygen via a face mask and maintained on 1.5–2% isoflurane in 100% oxygen for blood sample collection.

### Blood sample collection

All blood samples were collected from chemically immobilised animals. Beavers were positioned in dorsal recumbancy and blood samples were obtained from the ventral coccygeal blood vessels, 2–4 cm from the base of the tail as described by Greene *et al*. [22] and Eisele *et al*. [23].

Blood for serum biochemistry analysis was collected in 10 ml plain vials then the serum separated within two hours of collection after centrifugation at 10,000 rpm for 3 minutes. Where immediate analysis was not possible, serum was then frozen at -80°C until biochemical assays were undertaken. Blood for haematology analysis was collected in 5 ml vials containing potassium EDTA as anticoagulant and a fresh blood smear made for cytological analysis. All haematology samples were analysed within 1–2 days of blood collection after refrigerated storage.

### Haematology and Biochemistry

Haematological parameters were measured in 24 Eurasian beavers (leucocyte count, erythrocyte count, haemoglobin levels, mean cell volume, mean cell haemoglobin levels, mean cell haemoglobin concentrations and platelet count) using a Beckman-Coulter AcT 5 Diff. haematology analyser according to manufacturer’s guidelines (Beckman-Coulter UK Ltd). Differential white cell counts were performed manually based on a blood smear assessment of 200 leucocytes.

Total proteins were measured in 35 Eurasian beavers using the Biuret method with albumin, alpha, beta and gamma globulin levels being determined using agarose gel electrophoresis on a Helena Laboratories Rapid Electrophoresis analyser according to manufacturer’s guidelines (Helena Laboratories, Texas). All other biochemical parameters were analysed using a Randox Imola clinical chemistry analyser according to manufacturer’s guidelines (Randox Laboratories Ltd. London) in a total of 35 Eurasian beavers.

The differences in number of beavers tested between haematological parameters (N = 24) and biochemical parameters (N = 35) was due to rapid clotting of beaver blood in potassium EDTA anticoagulant making haematological testing in 11 of the beavers sampled impossible. The makeup of the beavers sampled for haematology was for Norwegian group 7 males and 5 females, and for the Bavarian group 6 males and 6 females. Both groups covered the complete age range of 6 months to 12 years

### Statistical Analysis

For each variable the mean, median, standard deviation and 90% reference range were derived to produce overall parameters for the Eurasian beaver. In addition, Welsh’s two-sample unequal variance t-test was used to compare the Norwegian and Bavarian populations and results of the 95% confidence interval test for differences in means were obtained using Minitab software version 16.2.3 and NCSS version 7 for bootstrap estimation. These differences were expressed as standardised effect sizes using the pooled standard deviation of the variable. Within the population sampled, Welsh’s two-sample unequal variance t-test was also used to compare male Eurasian beavers with female Eurasian beavers and to compare those under 3 years of age (sexually immature) with those over or equal to 3 years of age (sexually mature).

## Results

### Haematology

Haematological parameters for Eurasian beavers are shown in Table 1. Comparisons of Norwegian and Bavarian beavers are shown in Table 2.

**Table 1:** Overall range, mean, standard deviation (SD) and median for each haematological parameter for the Eurasian beaver (combined populations).

Parameter	5th-95th percentile	Mean	SD	Median
Red cell count (x10^12^/L)	3.30–4	3.69	0.25	3.7
Haemoglobin (g/dL)	11.83–14.29	12.98	0.98	12.85
Packed cell volume (L/L)	0.33–0.41	0.37	0.03	0.37
Mean cell volume (fl)	91–106	99.71	5.74	100.5
Mean cell haemoglobin (pg)	32.15–38	35.25	2.05	35
Mean cell haemoglobin concentration (g/dL)	34.15–36	35.25	0.60	35
Platelets (x10^9^/L)	149.6–308.25	216.5	49.86	209
White cell count (x10^9^/L)	6.87–13.43	10.45	2.09	10.80
Neutrophils segmented (x10^9^/L)	4.15–9.56	7.5	1.72	7.75
Neutrophils banded (x10^9^/L)	0–0.54	0.08	0.18	0
Lymphocytes (x10^9^/L)	0.92–4.24	2.43	1.04	2.25
Monocytes (x10^9^/L)	0.1–0.9	0.33	0.30	0.2
Eosinophils (x10^9^/L)	0–0.2	0.08	0.09	0.1
Basophils (x10^9^/L)	0	0	0	0

(N = 24).

**Table 2:** Summary of haematology values for relict Norwegian (N = 12) and Bavarian (N = 12) Eurasian beavers.

Parameter	Norwegian range 5th-95^th^ percentile	Bavarian range 5th-95^th^ percentile	Norwegian mean (SD)	Bavarian mean (SD)	Difference in means (95% confidence interval)	Two sample T test p-value	Standardised effect size between groups
Red cell count (x10^12^/L)	3.36–4.00	3.30–4.13	3.70 (0.230)	3.68 (0.298)	0.017 (-0.210, 0.243)	0.880	0.06
Haemoglobin (g/dL)	11.8–13.1	12.5–15.0	12.3 (0.473)	13.6 (0.968)	-1.30 (-1.96, -0.637)	0.001*	-1.71
Packed cell volume (L/L)	0.330–0.370	0.346–0.428	0.349 (0.0291)	0.385 (0.0144)	-0.0358 (-0.0557,-0.0160)	0.001*	-1.56
Mean cell volume (fl)	90.6–98.9	101.6–107.4	94.6 (3.18)	104.8 (2.12)	-10.2 (-12.6,-7.90)	0.000*	-3.77
Mean cell haemoglobin (pg)	32.0–35.0	35.6–38.0	33.5 (1.09)	37.0 (1.13)	-3.50 (-4.44, -2.56)	0.000*	-3.15
Mean cell haemoglobin concentration (g/dL)	35.0–36.0	34.0–36.0	35.2 (0.452)	35.2 (0.754)	0.00 (-0.53, 0.53)	1.000	0.00
Platelets (x10^9^/L)	135.2–302.2	173.3–298.2	213.4 (58.3)	219.6 (44.8)	-6.2 (-50.4,38.1)	0.774	-0.12
White cell count (x10^9^/L)	6.39–11.60	9.42–13.78	9.09 (1.76)	11.81 (1.55)	-2.72 (-4.12,-1.31)	0.001*	-1.64
Neutrophils segmented (x10^9^/L)	3.83–8.94	7.32–9.66	6.50 (1.86)	8.50 (0.974)	-2.00 (-3.27,-0.73)	0.004*	-1.35
Neutrophils banded (x10^9^/L)	0.000–0.645	0.000–0.000	0.167 (0.239)	0.000 (0.000)	-0.167 † (-0.283, -0.025)	0.034† *	-0.99
Lymphocytes (x10^9^/L)	1.12–3.86	0.86–4.08	2.208 (0.995)	2.64 (1.12)	-0.433 (-1.334,0.467)	0.328	-0.41
Monocytes (x10^9^/L)	0.100–0.245	0.200–0.990	0.142 (0.067)	0.517 (0.355)	-0.375 (-0.592, -0.158)	0.003*	-1.47
Eosinophils (x10^9^/L)	0.000–0.100	0.000–0.290	0.050 (0.0522)	0.1 (0.121)	-0.050 (-0.1314,0.0314)	0.209	-0.54
Basophils (x10^9^/L)	0.000–0.000	0.000–0.000	0	0	0	1	0.00

† denotes estimated by bootstrapping.

* significant at 5% level.

Significant differences (p< 0.01) were noted between the relict Norwegian and Bavarian beavers for haemoglobin levels, packed cell volume, mean cell volume, mean cell haemoglobin, white cell, and monocyte counts with significant differences with p< 0.05 for neutrophil (segmented and unsegmented) counts. In all of the above listed significant parameters the values for the Bavarian group were greater than those for the relict Norwegian group with the large standardised effect sizes (magnitude >3) being associated with mean cell volume and mean cell haemoglobin. There were no statistical significant differences in haematological parameters between different sexes (p<0.05) or between sexually immature (<3 years old) versus sexually mature (≥3 years old) (p<0.05) beavers sampled in this study.

### Serum biochemistry

Table 3 displays the serum biochemistry parameters determined for wild Eurasian beavers. Comparisons of Norwegian and Bavarian beavers are shown in Table 4.

**Table 3:** Overall ranges, means, standard deviations (SD) and medians for serum biochemistry parameters for the Eurasian beaver (combined populations).

Parameter	5th-95th percentile	Mean	SD	Median
Alanine transferase (IU/L)	28.7–49.9	36.83	7.28	36
Aspartate aminotransferase (IU/L)	60–93.2	74.8	12.59	73
Alkaline phosphatase (IU/L)	209.8–575.3	366.23	135.72	346
Bile acids (μmol/L)	3–6	3.54	1.13	3
Total bilirubin (μmol/L)	3.2–5.83	4.41	0.88	4.40
Total protein (g/L)	58.4–70	64.89	3.78	65
Albumin (g/L)	33.7–39	36.43	1.70	36
Alpha 1 globulin (g/L)	2.02–3.71	2.70	0.66	2.58
Alpha 2 globulin (g/L)	7.84–12.67	10.32	1.45	10.67
Beta globulin (g/L)	5.83–9.52	7.85	1.16	7.84
Gamma globulin (g/L)	4.57–10.22	7.38	1.81	7.28
Cholesterol (mmol/L)	1.87–3.86	2.71	0.65	2.70
Triglycerides (mmol/L)	0.45–1.71	0.99	0.49	0.96
NEFA (mmol/L)	740.7–1258.3	1006.89	164.67	986
Sodium (mmol/L)	129–140	133.57	3.37	133
Potassium (mmol/L)	3.87–5.53	4.44	0.58	4.20
Sodium:Potassium ratio	23.81–35.17	30.56	3.74	31.40
Chloride (mmol/L)	80.7–89	84.94	2.65	85
Urea (mmol/L)	3.14–9.06	5.99	2.02	6
Creatinine (μmol/L)	56.1–120.2	88.69	21.62	90
Total calcium (mmol/L)	2.17–2.8	2.54	0.19	2.60
Phosphorus (mmol/L)	1.20–5.22	2.88	1.65	1.50
Magnesium (mmol/L)	1.27–1.8	1.51	0.15	1.50

(N = 35).

**Table 4:** Summary of serum biochemistry values for relict Norwegian (N = 18) and Bavarian (N = 17) Eurasian beavers.

Parameter	Relict Norwegian Eurasian beaver range 5th-95^th^ percentile	Bavarian Eurasian beaver range 5th-95^th^ percentile	Relict Norwegian Eurasian beaver mean (SD)	Bavarian Eurasian beaver mean (SD)	Difference in means (95% confidence interval)	Two sample T test p-value	Standardised effect size between groups
Alanine transferase (IU/L)	28.8–52.6	29.4–45.8	37.5 (8.30)	36.12 (6.45)	1.38 (-3.73,-6.49)	0.585	0.19
Aspartate aminotransferase (IU/L)	60.8–93.1	58.0–92.8	74.7 (12.8)	74.9 (13.2)	-0.27 (-9.21,8.66)	0.951	-0.02
Alkaline phosphatase (IU/L)	215.7–599.8	204.8–573.2	352.6 (156.0)	380.7 (118.0)	-28.1 (-123.3,67.0)	0.550	-0.20
Bile acids (μmol/L)	3.00–6.15	3.00–4.00	3.94 (1.47)	3.12 (0.332)	0.827 (0.077,1.576)	0.032*	0.78
Total bilirubin (μmol/L)	2.99–5.95	3.44–4.94	4.56 (1.14)	4.24 (0.52)	0.320 (-0.293,0.933)	0.292	0.36
Total protein (g/L)	59.8–70.0	56.6–69.6	64.9 (3.61)	64.82 (4.17)	0.12 (-2.57,2.82)	0.928	0.03
Albumin (g/L)	33.8–39.0	33.8–38.2	36.7 (1.87)	36.1 (1.54)	0.605 (-0.572,1.781)	0.303	0.35
Alpha 1 globulin (g/L)	2.05–4.49	1.83–3.06	2.86 (0.80)	2.53 (0.45)	0.325 (-0.122,0.773)	0.148	0.50
Alpha 2 globulin (g/L)	7.65–11.14	10.1–12.8	9.35 (1.24)	11.34 (0.90)	-1.98 (-2.73,-1.24)	0.000*	-1.83
Beta globulin (g/L)	6.17–9.42	5.61–9.30	7.96 (1.27)	7.74 (1.10)	0.214 (-0.601,1.030)	0.596	0.18
Gamma globulin (g/L)	5.86–10.51	3.19–8.96	7.99 (1.75)	6.74 (1.74)	1.25(0.053,2.458)	0.041*	0.72
Cholesterol (mmol/L)	1.87–3.45	2.04–4.04	2.48 (0.56)	2.96 (0.68)	-0.487 (-0.016,-0.058)	0.027*	-0.78
Triglycerides (mmol/L)	0.51–1.55	0.44–1.51	1.03 (0.43)	0.94 (0.58)	0.083 (-0.268,0.434)	0.633	0.16
NEFA (mmol/L)	673.9–1353.1	956.0–1134.0	995.0 (25.0)	1019.2 (72.5)	-24 (-140.5,92.5)	0.672	-0.44
Sodium (mmol/L)	129.0–138.4	129.0–140.0	132.9 (3.16)	134.3 (3.64)	-1.41(-3.76,0.95)	0.233	-0.41
Potassium (mmol/L)	3.90–5.69	3.80–4.42	4.75 (0.67)	4.112 (0.21)	0.638 (0.291,0.986)	0.001*	1.29
Sodium:Potassium ratio	23.3–34.4	29.2–35.1	28.5 (4.02)	32.7 (1.87)	-4.24 (-6.41,-2.06)	0.000*	-1.35
Chloride (mmol/L)	79.8–88.1	81.0–89.0	84.8 (2.65)	85.1 (2.80)	-0.340 (-2.220,1.540)	0.715	-0.12
Urea (mmol/L)	5.47–10.01	2.88–7.02	7.35 (1.63)	4.56 (1.35)	2.791 (1.762,3.820)	0.000*	1.86
Creatinine (μmol/L)	89.5–123.4	51.8–86.2	106.3 (12.1)	70.0 (12.0)	36.33 (28.01, 44.66)	0.000*	3.01
Total calcium (mmol/L)	2.57–2.80	2.10–2.62	2.68 (0.11)	2.39 (0.16)	0.284 (0.187,0.380)	0.000*	2.07
Phosphorus (mmol/L)	1.20–1.50	3.28–5.54	1.35 (0.10)	4.51 (0.71)	-3.156 (-3.523,-2.789)	0.000*	6.22
Magnesium (mmol/L)	1.29–1.72	1.28–1.80	1.506 (0.147)	1.506 (0.171)	0.000 (-0.111,0.110)	0.996	0.00

* denotes significant at 5% level.

Significant differences (p<0.05) were noted between the relict Norwegian and Bavarian Eurasian beaver groups for bile acids, alpha 2 globulins, gamma globulin, cholesterol, potassium, sodium:potassium ratio, urea, creatinine, phosphorus and total calcium. The values were significantly greater for the Bavarian group versus the relict Norwegian group for alpha 2 globulins, cholesterol, sodium:potassium ratio and phosphorus. The reverse was true for bile acids, gamma globulin, potassium, urea, creatinine and total calcium, in which Norwegian beavers displayed significantly greater values than Bavarian. Large standardised effect sizes (magnitude >3) were associated with phosphorus and creatinine. There were no statistical significant differences in biochemical parameters tested based on differences in the sex (p<0.05) or between sexually immature (<3 years old) versus sexually mature (≥3 years old) (p<0.05) beavers sampled in this study.

## Discussion

This study is the first to our knowledge to provide baseline information on the haematology and serum biochemistry parameters and their variations in the Eurasian beaver.

Kitts *et al*. [15] investigated haematology of the North American beaver and concluded their results were of comparative values to other rodents. However, North American beavers did possess a lower red cell count than other rodents. The average diameter of the North American beaver erythrocyte is slightly larger than expected from other mammalian species, thought to be due to the semi-aquatic nature of this genus [15]. In the case of this study, the Eurasian beaver mean cell volumes are nearly double that of non-aquatic rodents such as rats (*Rattus norvegicus*) (upper range 64fl) and mice (*Mus musculus*) (upper range 57fl) [24] and are comparable with previously published volumes in North American beavers (83.2–118.8fl [15,19]) and in nutria (*Myocastor coypus*) another semi-aquatic rodent (84–102.5fl [25]). Red cell counts may be elevated in dehydrated animals, and reduced in those demonstrating blood loss or other causes of anaemia.

Whilst many parameters were found to be similar for both groups of beavers it is interesting to note that there are significantly different haematological results (p<0.05) between the relict Norwegian and Bavarian Eurasian beavers for some parameters. Haemoglobin levels have been shown to vary with the time of year in North American beavers, being higher in the autumn than the summer [15]. In this study, blood samples were collected at the same time of year ruling this out as a possibility for differences between the two location groups. Values for haemoglobin from both sets of Eurasian beavers in this study overlapped those of Kitts *et al*. [15] and were comparable to ranges for most other rodent species [26]. Elevated haemoglobin levels are often associated with dehydration and reduced levels with anaemia and liver disease.

Packed cell volumes (PCV) were slightly lower in the Eurasian beavers in this study compared with published ranges for the North American beaver (overall range 0.33–0.43 versus North American beavers of 0.4–0.46 [15]) or nutria (0.39–0.45 [25]). This indicates a lower cellularity of the blood although the total number of red cells for both populations of Eurasian beavers were within the lower end of the reported North American species range (3.7-5x10^12^/L [15]) and reported nutria range (3.9-6x10^12^/L [25]) suggesting this is related to the differences in mean cell volume seen. PCV, mean cell volumes (MCV) and mean cell haemoglobin concentration (MCHC) were also significantly greater in the Bavarian versus the relict Norwegian Eurasian beavers. It has long been known that these parameters vary between different species so it is interesting to note these differences within a species and may suggest that there are population differences between the relict Norwegian and Bavarian Eurasian beavers [27]. The age of the animal can also increase MCV in other species with very young animals often possessing larger MCVs [27]. However the age distribution for both populations was similar with no neonates included in the study and no evidence of significant differences were noted in this parameter between sexually immature and sexually mature animals. Iron deficiency anaemia can lead to a decrease in MCV, MCHC and haemoglobin levels [27]; but no clinical anaemia was noted in any individual included in this study suggesting that this also could not account for the differences.

White blood cell counts were again comparable to that previously published in North American beavers (7–18.2x10^9^/L [15]) and nutria (9.9–13.1x10^9^/L [25]). Between the two location groups in this study there was again a significant difference with the Bavarian group demonstrating a greater white cell count than the relict Norwegian. It should be noted that in some rodents such as rats and mice, significant diurnal variation is seen in leukocyte levels (increases during the light phase and decrease during the dark phase) but it is not known whether the same occurs in beavers [28]. Other causes for variation in white cell counts include infection, different handling techniques, neoplasia and stress [27]. Bacterial and fungal disease usually result in an overall increase in leukocyte levels and a left shift towards band forms of neutrophils with an increase in the neutrophil and in chronic cases of disease, monocyte counts. Immune stimulation and viral diseases may result in an increase in lymphocyte counts, although chronic antigenic depletion may also be seen with a reduction in lymphocyte numbers. Stress may result in an overall increase in the leucocyte, neutrophil and monocyte count but a relative reduction in the lymphocyte count (lymphopaenia). The process for collection was the same in both groups, so it is unlikely that stress or handling techniques could account for the differences. Blood borne neoplasia could also be ruled out as no evidence of neoplastic cells could be seen. Infection however is more difficult to rule out based on purely a clinical examination and it is highly possible that the ‘left shift’ seen with band forms of neutrophils in individuals in the Norwegian beavers may indicate a low level of inflammatory disease. This is however contrary to the overall white cell count which was actually lower in the relict Norwegian group. The population of monocytes was also significantly greater in the Bavarian group. Monocytosis may be seen in chronic active inflammatory disease, in conjunction with a leucocytosis and often neutrophilia [27]. Clinically, this was not apparent in individuals of this group but could account for this variation as subclinical disease, or it may again be a genuine variation between the two location groups.

Total protein levels in Eurasian beavers in this study fall within the ranges published for North American beavers, although as with albumin levels seasonal variations were noted in the North American beaver study which cannot be assessed in this study [16]. In comparison with nutria they were at the lower end of their range (nutria total protein ranges 63–89g/L [25]). Blood proteins play an important role in maintaining overall oncotic potential preventing significant extravasation of fluid from the circulation and so again it is not surprising that there is no significant variation between the two location groups for total proteins or the overall levels of the globulins or albumin. However within the different subset of globulins analysed, one significant difference was observed in the alpha 2 globulins with the Bavarian Eurasian beaver group demonstrating higher levels than the relict Norwegian Eurasian beaver group. Alpha 2 globulins are manufactured in the liver and include: alpha 2 macroglobulin whose levels may be raised with the nephrotic syndrome, nephritis, acute inflammatory disease and chronic active hepatitis; and very low density lipoproteins which may also be elevated with the nephrotic syndrome, diabetes mellitus and hypothyroidism [12]. These acute phase proteins found in the alpha and beta globulin sections of the protein electrophoretogram can be used therefore to assess a non-specific inflammatory response to disease or trauma or organ failure and so are useful in assessing animal health. Gamma globulins contain the main immunoglobulin IgG in mammals and may be used to assess humeral immunity stimulation by an antigen such as a virus and so can be useful in assessing disease exposure [12]. Values here were similar between the two subgroups.

Creatinine and urea are often used as an indicator of renal function in mammals with elevations in both parameters being seen in the latter stages of renal failure [12, 29]. It is interesting to note that there is a statistically significant difference between the two location groups studied here with the relict Norwegian group being greater than the Bavarian for both parameters. This is an important feature to note when attempting to interpret results in any renal health assessment. Values are similar to published values for nutria for creatinine but lower than nutria for urea [25].

Stevenson *et al*. [16] study demonstrated high levels of serum cholesterol in North American beavers. The beavers in that study were wild caught North American beavers but then retained in captivity for the study and so these high cholesterol levels may reflect the captive diet rather than the true cholesterol readings of wild North American beavers. In this study, cholesterol levels for Eurasian beavers were largely within the range published for many rodents including nutria [25, 26, 29]. There is a difference between the two location groups studied here with the Bavarian group being greater than the relict Norwegian at p<0.05, but not at p<0.01. This could be a reflection of the different herbage present in the two locations as cholesterol levels can be influenced by diet in animals with access to high levels of saponins (e.g. as found in clovers and members of the pea family) these have been shown to reduce cholesterol absorption levels. Genetic differences may also alter cholesterol levels as is seen in humans and other species [30]. Interestingly, triglyceride and NEFA levels were not significantly different at p<0.05 or p<0.01. NEFA and triglyceride levels are similar to those reported in other rodents and are directly associated with dietary levels maybe suggesting again a genetic difference in the cholesterol levels seen between the two groups [26, 29, 30].

Elevations in liver leakage enzymes alanine transferase (ALT) and aspartate transferase (AST) indicate hepatocellular damage in many mammals [12]. In rodents, AST is more liver specific but is also found in skeletal muscle [29]. Values for both enzymes were similar to those published for a wide range of rodent species [31] and slightly lower than that published for nutria [25]. Bile acid levels are often used as a liver function test with fasting values of <20umol/L being quoted as normal for most mammals [32]. Elevations in fasting animals indicate liver dysfunction [32]. Values here were clearly unlikely to be fasting results, but were nonetheless low with no difference between the two location groups tested. In rodents bilirubin is the end product of haemoglobin metabolism, and so when elevated can be used to indicate liver damage or haemolysis [29]. Values determined here were similar to those reported for other rodents [31].

Alkaline phosphatase (ALKP) is a leakage enzyme found in bone, the intestines and the liver predominantly [32]. Elevations can therefore occur due to bone, intestinal or liver disease, or due to bone growth in young animals. Values for the Eurasian beaver in this study were similar to those reported for other rodents [31] including nutria [25]. Electrolytes sodium and chloride values are also similar to that reported for nutria and other rodents however potassium levels varied significantly between the two location groups studied with the relict Norwegian group being greater than the Bavarian Eurasian beaver group. In all mammals, physiologically normal values for potassium do not exceed 7.5mmol/L as cardiac dysfunction occurs, or become less than 2.5mmol/L due to muscular dysfunction [33]. The values in this study fit within this range. It is possible with haemolysis for elevation in potassium levels to occur, and serum has higher levels of potassium than plasma. However handling and processing of the samples was the same for both groups making these less likely reasons. Potassium loss due to renal or gastrointestinal disease is unlikely as no other signs of disease were noted in the animals included in this study. Variations in the intake of potassium due to differing diets however is a more likely possibility.

Diet may also account for the significantly increased total calcium and lower phosphate levels in the relict Norwegian versus the Bavarian Eurasian beavers. Calcium:phosphorus ratios often follow an inversely proportionate relationship and again this may account for the results seen here. Calcium and phosphate levels reported here are within the range for other rodents and mammals [31]. Elevated phosphorus levels may also be seen in cases of advanced renal failure [29]. Lowered calcium levels may be seen in diets deficient in calcium, high in phosphorus or containing compounds such as oxalates (e.g. beet leaves) or phytates (e.g. grains) which bind ionised calcium in the gut and prevent its absorption.

Magnesium is an important micromineral aiding in the homeostatic mechanisms which maintain serum calcium and potassium levels with deficits often resulting in seizures, weakness, and unresolving hypocalcaemia and hypokalaemia [30]. Results here tally with the narrow physiological range from a variety of other mammals [30].

The data from this study could be used to assess and monitor the health of captive animals and other wild populations. This will have significant effects when assessing the compatibility of reintroduced beavers to new habitats. Currently in the UK this is of even greater significance as not only has a recent trial reintroduction been completed in Knapdale, Scotland, but wild-living populations of beavers have now been found throughout in other parts of Britain, most notably in South-West England and Scotland, numbering in their hundreds in some cases. The beaver has been shown to be a potential intermediate host for significant pathogens such as *Giardia* spp., *Franciscella tularensis* and the tapeworm *Echinococcus multilocularis* and as such assessing the health of any wild, captive or beavers of unknown origin is important for human health as well as for reintroduction assessments [34, 35]. Changes in blood chemistry values can be used in the diagnosis of diseases [29]. However, when establishing blood parameters for wild populations, capture methods must be taken into account as these can cause significant differences between reference values [36, 37] as will diet and other environmental factors [29].

Further studies should sample a larger number of individuals, from both wild populations and captive collections across Europe. The influence of habitat condition (e.g. free-living vs. captive) and habitat quality is another area requiring further research. Correlating blood parameters with known survival outcomes could generate fitness predictors for demographic class or populations.

## Supporting Information

S1 Table Total beaver blood results. (XLSX)

S2 Table Norway versus Bavarian blood results. (XLSX)
